# The Correlates of Post-Traumatic Stress Disorder in Ambulance Personnel and Barriers Faced in Accessing Care for Work-Related Stress

**DOI:** 10.3390/ijerph19042046

**Published:** 2022-02-11

**Authors:** Itumeleng Ntatamala, Shahieda Adams

**Affiliations:** Division of Occupational Medicine and Centre for Environmental and Occupational Health Research, School of Public Health and Family Medicine, University of Cape Town, Cape Town 7925, South Africa; shahieda.adams@uct.ac.za

**Keywords:** paramedic, ambulance personnel, occupational, work-related stress, PTSD, post-traumatic stress, barriers

## Abstract

We investigated factors associated with increased risk for post-traumatic stress disorder (PTSD) in ambulance personnel and the barriers faced in accessing support for work-related stress (WRS). A cross-sectional study of 388 ambulance personnel used self-administered questionnaires to assess for PTSD and level of occupational stressors: Impact of Event Scale-Revised, Emergency Medical Services (EMS) Critical Incident Inventory, EMS Chronic Stress Questionnaire, SF-36 Quality of Life and the Connor–Davidson Resilience Scale. The prevalence of PTSD in the study population was 30%. The participants were predominantly female (55%), with a median age of 38 (IQR; 31–44) years. PTSD was associated with smoking (OR = 1.76, 95% CI: 1.05–2.95), illicit drug use (OR = 16.4, 95% CI: 1.87–143.86) and problem drinking (OR = 3.86, 95% CI: 1.80–8.23). A self-reported mental health condition (OR = 3.76, 95% CI: 1.96–7.21), being treated for a medical condition (OR = 1.95, 95% CI: 1.22–3.11), exposure to critical incident stress (OR = 4.27, 95% CI: 2.24–8.15) and chronic WRS (OR = 4.46, 95% CI: 1.93–10.31) were associated with PTSD risk. Barriers to seeking help included concerns that services were not confidential and the negative impact on the participant’s career. The increased levels of WRS, strong associations with substance use and barriers to accessing care offer starting points for workplace interventions to reduce the impact of PTSD in ambulance personnel.

## 1. Introduction

Exposure to occupational stressors, such as verbal and physical assault and responding to traumatic scenes including motor vehicle accidents or critically ill patients, has been linked to the development of stress reactions such as post-traumatic stress disorder (PTSD) and other trauma-related conditions in frontline emergency workers [1,2,3,4,5,6,7].

Studies have found an increased PTSD prevalence in ambulance personnel compared with the general population and other emergency workers. A pooled PTSD prevalence of 10% was found in emergency workers compared to 1.3–3.5% in the general population [8]. PTSD prevalence was highest among ambulance personnel (14.6%, 95% CI: 8.80–20.30) compared to fire fighters (7.30%, 95% CI: 3.60–11.00) and police officers (4.70%, 95% CI: 1.20–8.30). Others have reported an estimated PTSD prevalence of 11% among ambulance personnel worldwide, which was higher than that of the general population [9]. Possible explanations for increased PTSD prevalence include the fact that ambulance personnel respond to a greater number of calls than other emergency workers, have greater personal contact with patients and their families, have more sustained work pressure when on duty and often work with limited resources in strained health systems [8,9,10,11,12]. Cross-sectional studies have identified demographic factors such as younger age, female gender, educational level, marital status and living alone as correlates for PTSD in ambulance personnel [13,14,15,16]. Other significant correlates include the frequency and intensity of exposure to acute and chronic stress [17,18,19], lack of social support from colleagues and management [12,20], pre-existing psychiatric illness and poor coping style [5,16].

The prevalence of PTSD in South African ambulance personnel was reported as 6.67% (in 2005) and 16% (in 2014) [11,12]. Ward et al. [6] found that South African ambulance, fire and sea rescue service workers experienced more exposure to critical events and suffered higher rates of general psychopathology compared to workers in high-income countries.

Despite evidence of increasing exposure to critical events, there is a paucity of research in South Africa documenting trends in the prevalence of PTSD and factors associated with its development in ambulance personnel. Little is also known about what barriers they face in accessing support for work-related stress. This study aimed to determine factors associated with an increased risk for PTSD and the preferred supportive measures for work-related stress (WRS) in ambulance personnel in the Western Cape Province, South Africa.

## 2. Materials and Methods

### 2.1. Study Design, Population and Sampling

A cross-sectional study of ambulance personnel employed by the Western Cape Department of Health situated at each of 50 ambulance bases was conducted between 15 November 2019 and 17 January 2020. An electronic questionnaire was distributed to all of the ambulance personnel in operational (providing direct clinical care) and support service roles. Both electronic and hard copy versions of the questionnaires were provided based on the participant’s choice. No exclusion criteria were applied in this study. All of the participants were required to provide informed consent prior to participating in the study. The study was approved by the University of Cape Town’s Human Research Ethics Committee (HREC 517/2019).

### 2.2. Measurements

#### 2.2.1. Author Questionnaire

The author questionnaire gathered self-reported sociodemographic data including age, gender, language, level of education, marital status, living status and occupational data such as ‘job category, length of service, location of work’. Mental health and medical history data were obtained, including ‘own/family history of mental health diagnosis, treatment for medical or mental health condition’. Smoking, alcohol, illicit and prescription drug use history was also solicited. The CAGE questionnaire was used to assess problem drinking (score of ≥2/4 indicative of problem drinking). Questions on barriers faced in seeking help for work-related stress and preferred sources of support were included.

#### 2.2.2. Impact of Event Scale-Revised (IES-R)

The Impact of Event Scale-Revised (IES-R) was used to assess post-traumatic stress symptoms experienced (during the past seven days) in relation to the most troubling critical incident encountered in the line of duty in the past 6 months. This validated and widely used 22-item scale contains questions that cover three PTSD symptom clusters [21,22]. Symptoms are scored on a five-point Likert scale (0-4) in response to the most troubling critical or traumatic event/s experienced in the workplace. The higher scores obtained indicated greater symptomatic stress, and a cut-off score of above 35 was used as indicative of a PTSD ‘case’ [22].

#### 2.2.3. Connor-Davidson Resilience Scale (CD-RISC)

The validated 10-item Connor-Davidson Resilience Scale (CD-RISC) was used to measure resilience in the ambulance personnel. The participant’s adaptive behaviours in stressful situations were used to identify resilient characteristics and these were scored on a 5-point Likert scale. Higher scores indicate greater resilience. The CD-RISC scale has been noted as one of the most reliable and efficient measures of resilience with an internal consistency of between 0.7 from 0.9 [23]. Written permission to use the scale was obtained from the authors.

#### 2.2.4. EMS Critical Incident Inventory (CII)

The Emergency Medical Services (EMS) Critical Incident Inventory (CII) developed by Ward et al. [6] and Donnelly and Bennett [24] was used to assess exposure to critical incidents encountered in the line of duty and to obtain an indication of the frequency of exposure in the past 6 months. The activities encountered during ambulance work are contained in the CII.

#### 2.2.5. EMS Chronic Stress Questionnaire (EMS-CSQ)

The EMS Chronic Stress Questionnaire (EMS-CSQ) is a validated tool used to assess the exposure to and perceptions of chronic stress experienced by ambulance personnel [7,25]. The tool assesses both organizational and operational types of chronic workplace stress, with each scale consisting of 10 questions. It defines operational stress as that associated with structural elements of working in EMS such as shift work, risk of being injured in the line of duty and fatigue. Organizational stress includes factors associated with organizational culture, such as conflict with supervisors and staff shortages.

#### 2.2.6. SF-36 Quality of Life Questionnaire (SF-36 QOL)

Reduced quality of life and interference with occupational functioning due to emotional problems such as feeling depressed, anxious or stressed was measured using three questions from the SF-36 Quality of Life questionnaire (SF-36 QOL). This validated tool is widely used to measure general health status in emergency workers and the general population [26,27].

### 2.3. Statistical Analysis

Stata 14.0 statistical computer software (StataCorp, College Station, Texas, USA) as was used for the analysis of all of the data collected. The main associations of interest were between the explanatory variables (socio-demographic, occupational and environmental risk factors, general and mental health status) and post-traumatic stress disorder as an outcome variable. Bivariate analysis was then undertaken to examine the correlation between post-traumatic stress symptom scores with various workplace and environmental risk factors. These variables were then analyzed using unadjusted logistic regression with a calculation of the odds ratio (OR) followed by multivariate regression analysis, with adjustment for age, gender and educational level. To further explore the relationship between PTSD and continuous variables (resilience, chronic stress and critical incident stress scores), three tertiles of these variables were created (high, moderate and low) while four categories were created for age and SF-36 QOL scores.

## 3. Results

Of the 2000 distributed questionnaires, 478 were returned representing a 24% response rate. However, two were duplications and 88 were incomplete and could not be used in the analysis. Only 388 entries were therefore analyzed in this study.

### 3.1. Demographic and General Health Qualities of Participants

The demographic characteristics of the study population are outlined in Table 1. The participants were predominantly female (55%), with median age 38 (IQR: 31–44) years and most had a professional qualification (83%). The majority lived with family or friends (79%).

A third of participants (30%) were current smokers while half (52%) currently used alcohol (Table 2). Of those who currently used alcohol (*n* = 200), 27% had problem drinking based on their CAGE score. Males had significantly more problem drinking than females (32.03% vs. 18.06%; *p*-value = 0.03). Only 3% of all of the participants indicated current use of illicit/non-prescription drugs.

### 3.2. Occupational History

Most of the participants worked in operational services (71%) compared to support services (Table 1). Half worked in a rural setting (53%). The median years of employment in current role was 7.7 (IQR: 3.4–12.2) while the overall median years employed in a health environment was 9.9 (IQR: 5.8–15). A significant but moderate positive correlation (Spearman rho 0.52, *p* < 0.001) existed between participant age and employment duration in their current role. A similarly positive yet strong correlation (Spearman rho 0.69, *p* < 0.001) existed between participant age and employment duration in the health sector. A quarter (25%) of the participants reported changing jobs in the past five years primarily for better job prospects, fewer for medical reasons.

### 3.3. Mental Health Status and Work-Related Stress Management

The self-reported prevalence of ever being diagnosed with a mental health condition was 11%, with 7% reporting currently being on treatment for a mental health condition (Table 2). Based on the IES-R questionnaire, the prevalence of PTSD in all of the participants was 30.41%; females had a higher prevalence (35%) compared to males (27%), but this difference was not statistically significant (*p*-value = 0.085). PTSD prevalence in operational staff (30%) was also not significantly different (*p*-value = 0.853) to that of support services staff (31%) (Table 3).

Forty percent of participants reported having had emotional problems with regular work in the preceding 4 weeks. Substances used by participants to manage WRS include cigarette/tobacco smoking (27%), drinking alcohol (11%), illicit drug use (4.13%) and the use of prescription medication (17%). The majority had not received any specific training on how to manage WRS (69%) or on what support services were available (69%). The greatest barrier encountered in seeking help for WRS is the fear that the services provided by the employer were not confidential (38%) and that the participant’s career would be negatively affected (23%). Preferred sources of support for participants include a family member or friend (63%), a spouse or partner (56%) and a spiritual leader (41%) while the least preferred (20%) is a trade union or labour representative (Table 4). The participants recommended various ways in which services for WRS could be improved (Table 3), including training for staff to recognize when they are stressed (68%) and more supportive management (63%), with the least preferred method being telephonic counselling (20%).

### 3.4. Occupational and Environmental Risk Factors for PTSD

Bivariate analysis was undertaken to examine the correlation between post-traumatic stress symptom scores with various workplace risk factors. These associations were all found to be statistically significant (Table 5). Increasing PTSD scores had a significant but moderate positive correlation (Spearman rho 0.56, *p*-value < 0.001) with chronic workplace stress and a weak positive correlation (Spearman rho 0.34, *p*-value < 0.001) with critical incident stress. A significant but weak negative correlation (Spearman rho–0.25, p < 0.001) was found between PTSD and resilience, and a similar weak negative correlation (Spearman rho–0.22, *p*-value < 0.006) was found between PTSD and quality of life.

Unadjusted logistic regression analysis was performed (Table 6). PTSD was associated with working in operational services (Health-Net) (OR = 6.00, 95% CI: 1.32–27.10), needing to smoke to manage WRS (OR = 2.13, 95% CI: 1.33–3.41), needing to use alcohol to manage WRS (OR = 3.55, 1.87–6.75), alcohol misuse (OR = 2.85, 95% CI: 1.50–5.44), current illicit drug use (OR = 4.18, 1.20–14.58), feeling the need to use illicit drugs (OR = 4.07, 95% CI: 1.44–11.48) and the use of prescription medication to manage WRS (OR = 4.63, 95% CI: 2.65–8.09). Working in a rural area was found to be protective against PTSD (OR = 0.61, 95% CI: 0.40–0.95).

Participants with a self-reported mental health condition were more likely to have PTSD (OR = 3.76, 95% CI: 1.96–7.21), including those currently on treatment for a medical condition (OR = 1.95, 95% CI: 1.22–3.11). Those who had emotional problems with regular work in the past 4 weeks were also more likely to have PTSD (OR = 5.37, 95% CI: 3.36–8.59). PTSD was positively associated with chronic workplace stress, with the strength of association greater in those with moderate (OR = 4.71, 95% CI: 2.16–10.30) to high (OR = 20.03, 95% CL: 9.33–43.03) levels of chronic workplace stress compared to those with low WRS. Furthermore, PTSD was associated with critical incident stress; those with the highest critical incidence stress displayed greater odds of being diagnosed with PTSD (OR = 3.19, 95% CI: 1.88–5.40). PTSD was negatively associated with increased quality of life (OR = 0.38, 95% CI: 0.15–0.94) and increased resilience score (OR = 0.26, 95% CI: 0.14–0.48)).

Multivariate regression analysis was computed (Table 6), with adjustment for age, gender and educational level. PTSD was associated with being a current smoker (OR = 1.76, 95% CI: 1.05–2.95) (previously non-significant) and feeling the need to smoke to manage WRS. Some associations between PTSD and various risk factors were strengthened following adjusted analysis. These included: the need to use alcohol to manage WRS (OR = 6.37, CI: 2.93–13.85), alcohol misuse (OR = 3.86, 95% CI: 1.80–8.23), current illicit drug use (OR = 16.4, 95% CI: 1.87–143.86) and feeling the need to use illicit drugs (OR = 5.99, 95% CI: 1.74–20.62). The association between PTSD and working in operational services (Health-Net) was no longer significant. However, PTSD was now associated with working in the call centre (OR = 4.04, 95% CI: 1.07–15.21). Increased exposure to chronic and critical workplace stress continued to be positively associated with PTSD while increasing levels of quality of life and resilience were protective.

## 4. Discussion

The prevalence of PTSD in this study was found to be 30%, which is higher than that reported for the South African general population (2.3% lifetime prevalence) [28] and reported globally (1.0–4.3%) [29]. It is also higher than estimated by previous studies conducted in the Western Cape, which found a prevalence of 6.7% in ambulance personnel (*n* = 99) and 16% (n = 131) in paramedic trainees [11,12]. When compared to other lower-middle-income countries (LMICs), PTSD prevalence in this study is higher than the 13.6% reported for Egyptian paramedics (*n* = 140) and 15% in Brazilian ambulance workers (*n* = 234) [30,31]. Systematic reviews and meta-analyses from studies conducted in high-income countries (HICs) report a prevalence as low as 11% [9] and as high as 20% among ambulance personnel [32].

The higher prevalence found in this study can be explained by differences in study population, context and methodology. In the Western Cape Province, as of 2014, an escalation in criminal attacks was experienced by ambulance personnel (including threats, verbal/physical assaults, robbery/theft, random stoning), with likely increases in chronic stress and critical incident stress exposure [33]. To mitigate the potential impact of these traumatic exposures on ambulance personnel, certain areas within the Cape Town Metropole were designated as ‘Red Zones’, requiring a police escort be made available before ambulance personnel go into an area to assist patients [33]. Previous studies undertaken before 2014 used a different PTSD scoring scale and had relatively smaller sample sizes than this study. Given the escalation in attacks on paramedics since 2014, the participants in this study may be at greater risk for PTSD and more likely to have concerns regarding PTSD and a need to report their symptoms compared to non-participants [13].

Of note in the current study is that the PTSD prevalence in operational staff (30%) consisting of ambulance personnel on the road was not significantly different (*p*-value = 0.853) to that of support services staff (31%) consisting largely of call center workers (*n*= 88) and managers/staff in various administrative roles (*n* = 33). The similar PTSD prevalence may be because of the relocation or accommodation of operational staff previously exposed to trauma to a call centre or management role due to mental or physical health or reasons of incapacity. The lack of skills in handling the secondary trauma related to working (telephonically) with families or patients in distress may also be independently associated with PTSD status. It has been noted that, although removed from the scene of the incident, emergency service call-takers and dispatchers experience similar mental health challenges as front-line ambulance personnel. [34].

Current mental health problems have been demonstrated as a predictor of PTSD [35,36]. Kerai et al. [16] found that emergency personnel with anxiety and depression had higher levels of post-traumatic stress symptoms. Those with a pre-existing mental health condition may be predisposed to developing PTSD in the presence of chronic and acute stress events associated with first responder work [2,21,37]. Participants who reported having a mental health condition were more likely to have PTSD and more likely to report emotional problems in the past 4 weeks with role limitations due to emotional problems. These findings were consistent with those reported in Brazilian paramedics [31], with the mean (SD) QOL score for role limitation due to emotional problems in our study being 37.2 (IQR: 38.42), which was lower than that for Brazilian paramedics with PTSD, which was 47.2 (IQR: 33.21). This indicates an even poorer quality of life in our population. Poorer quality of life score was also associated with increased odds of PTSD diagnosis. Participants with PTSD were also more likely to report being on treatment for a medical condition; this is a phenomenon also observed in Brazilian paramedics with PTSD as they had more medical visits and hospital admissions in the previous 12 months compared to those without PTSD.

Increased levels of exposure to chronic and critical incident stress were significantly associated with PTSD diagnosis, with those exposed to the highest levels having greater odds of being diagnosed compared to those with lower exposures, reflecting a dose–response relationship. The mean critical incident score (27.7) found in this study was higher than that previously found (20.59, 95% CI: 19.11 to 22.06) in this population [6,24], which is indicative of not only high exposure but a possible increase in critical incident stress over time. Chronic organizational and operational stress both individually and in combination are significant correlates of PTSD in this population. The mean (SD) operational stress score of 33.8 (15.27) was comparable to that found by Donnelley et al. [7] of 31.4 (12.1); while the mean (SD) organizational stress score of 42.5 (16.06) vs. 34.8 (13.5) was higher (13.5). These findings demonstrate high chronic stress exposure in the population surveyed and importantly highlight the potential impact organizational factors such as staff shortages have on PTSD prevalence. Staff shortages, for example, could result in ambulance personnel experiencing more frequent exposure to critical incidents and resultant increases in chronic stress levels. In this study, however, individuals who worked in a rural area were less likely to develop PTSD. This may reflect a workload with less exposure to chronic stress or less critical incident stress events compared to those working in urban settings.

The use of substances to manage or cope with WRS deserves further exploration. In this study, the need to smoke, drink alcohol and to use illicit drugs to manage WRS was associated with PTSD. Participants displayed high rates of problem drinking (27% overall, 32.03% in males and 18.06% in females). This was higher than that for paramedic trainees in the Western Cape (23%) and the general population (16% in men and 3% in women) [12,38]. The use of prescription drugs such as anxiolytics and antidepressants by 17% of the participants to manage WRS was also significantly associated with PTSD. This was higher than the 5.6% personal medication use reported in emergency medical service personnel (n = 518) in Pakistan [16]. While it is not clear whether the substance use is because of PTSD or preceded it, this places participants at risk of dual psychiatric diagnoses (substance use disorder as well as PTSD), which is more difficult to manage and has greater morbidity associated with it [17,21].

Participants indicated that they are most likely to seek help/support for WRS from a spouse or partner (62.50%) and family member or friend (55.94%), which highlights the important role that family and friends can play when formulating interventions aimed at reducing WRS. Canadian paramedics were similarly more likely to seek support from a family member or friend (81.4%) and a regular work partner (73.2%) [7]. Surprisingly, however, there was a lower likelihood of seeking support from employer-provided services, with only 25% of participants (compared to 38.6% in Canadian paramedics) likely to use such a service. Various barriers to accessing support included fear that services are not confidential and that participant’s career will be negatively affected. Difficulty in getting time off to access care and a lack of finances/medical aid are other barriers faced.

The participants identified the need for more training of staff in recognizing WRS, having a more supportive management, addressing staff shortages and provision of counseling on the work premises as preferred measures to help reduce WRS. These correlate with the chronic organizational stressors already discussed and highlights the extent to which these are concerning to the participants. Of note is that the majority (69%) reported having not received any specific training on how to manage WRS or on what services are available (69%). Minnie et al. [5] also found that 72% of ambulance personnel noted having received little or no training on managing the emotional effects of exposure to traumatic effects. Promoting resilience in ambulance personnel could also be helpful in mitigating the challenges related to the work of ambulance personnel [39], as reflected in a negative correlation between PTSD and resilience scores. Those with the highest resilience scores were less likely to be diagnosed with PTSD than those with lower scores. Similar studies have shown that lower resilience was significantly associated with PTSD status [12,39]. While resilience training helps equip emergency workers to deal with traumatic events, other interventions, such as access to psychiatric screening by trained mental health professionals for earlier detection of PTSD symptoms and provision of evidence-based treatments, should be considered [39,40,41]. Broader organizational and operational concerns raised such as a supportive work environment and follow-up of at-risk workers should be addressed to help prevent the development of PTSD from work-related trauma [40,41].

### Limitations

The study had several limitations. The findings are based on 388 respondents from a workforce of 2000 as it relied on voluntary participation. Ambulance personnel who chose not to participate may be different to those who did, which may alter the overall PTSD prevalence obtained, a reflection of selection bias. Reasons for lack of participation include possible stigma relating to a mental health topic and ‘response fatigue’ as other surveys were being conducted during the time of this study. Mistrust and concerns that the questionnaire would not be confidential or would negatively affect one’s work prospects may also be a contributing factor. However, this was mitigated by meeting trade union representatives and staff at ambulance bases to explain the study methodology and data handling concerns.

Not all of the potential risk factors for PTSD were assessed to help keep the questionnaire to an acceptable length. Questions on childhood trauma, the full SF36 quality of life questionnaire and other mental health screening tools could not be included. Due to resource limitations, the study was unable to use the gold standard test for the diagnosis of PTSD (clinician-based diagnostic interview); however, validated PTSD screening tools were used. Social desirability bias may have occurred when participants responded in a socially acceptable manner (e.g., underreporting mental health symptoms and substance use), which could have led to reduced study estimates. Recall bias may also have arisen as the questionnaire required participants to rate symptoms based on a traumatic event that was experienced up to 6 months earlier. There was no control group from a general population whose findings could be compared to those of the ambulance personnel. Further, studies of occupational groups are vulnerable to the healthy worker effect, which may have led to an underestimate of PTSD as those severely impaired by PTSD may have left their employ, leaving a generally healthier population behind. The cross-sectional study design further limits the inference of causal associations as data on both exposures and outcomes were collected simultaneously. Concordance with the findings from other studies and biological plausibility do, however, suggest that significant associations may be reflecting potential risk factors implicated in the development of PTSD in this population.

## 5. Conclusions

PTSD prevalence was markedly higher in ambulance personnel than in the general population, reflecting an increased risk in this occupational grouping. This is a cause for concern. Those with a mental health condition who were receiving treatment for a medical condition, who self-reported substance use and abuse, who were working in an urban area or who were exposed to moderate to high chronic and critical incident stress were at increased risk. Support from family and friends including co-workers could form an integral part of interventions designed to support ambulance personnel. Greater attention needs to be paid to the barriers that have been identified as limiting access to available support for work-related stress. In this regard, efforts should be aimed at ensuring access to confidential and acceptable mental health services that are provided in a non-punitive manner without leaving workers out of pocket. The high rates of organizational and work-related stress experienced by this population and the strong associations between PTSD and substance use offer starting points for interventions aimed at reducing the burden and impact of PTSD.

Further studies using clinician-based diagnostic interviews are recommended and research into evidence-based methods of screening and managing ambulance personnel for PTSD and the development of appropriate interventions and protocols for lower-middle-income countries context are needed.

## Figures and Tables

**Table 1:** Demographic and occupational qualities of participants (*n* = 388).

Participant Characteristics	Frequency (*n*)	Percentage (%)
**Sex**		
Male	175	45.10%
Female	213	54.90%
**Age**		
Age, years (*n* = 339)	38 (31–44)	
**Home language**		
Afrikaans	178	45.88%
English	122	31.44%
IsiXhosa	84	21.65%
Other	4	1.03%
**Level of education**		
Basic (Grade 1–12)	35	9.02 %
Certificate	284	73.20%
Diploma	45	11.60%
Degree	24	6.19%
**Marital status**		
Never married	172	44.33%
Married	174	44.85%
Divorced or separated	36	9.28%
Widowed	6	1.55%
**Place of living**		
Rural	153	39.43%
Urban	235	60.57%
**Current living status**		
Alone	81	20.88 %
Family or friends	307	79.12%
Occupational status		
Professional health qualification	322	82.99%
**Job category**		
**Operational services**	277	71.39
Ambulance Services	233	60.05%
HealthNet (Non-Emergency Transport)	19	4.90%
Rescue Services	25	6.44%
**Support services**	111	28.61
Call Centre	88	22.68%
Managers, Admin, Finance & Other	23	5.93%
**Years employed in current role (*n* = 383)**	7.7 (3.4–2.2)	
**Years employed in a health environment (*n* = 383)**	9.9 (5.8–15)	
**Place of work**		
Urban	184	47.42%
Rural	204	52.58%
**Job/role change at work in the past 5 years**	98	25.26%
**Average hours worked per week**		
30–40 h	122	31.44%
41–50 h	121	31.19%
51–60 h	130	33.51%
61–70 h	15	3.87%
**Monthly salary (South African Rands)**		
ZAR 0–10,000	41	10.57%
ZAR 10,001–20,000	238	61.34%
ZAR 20,001 and above	109	28.09%

Data are presented as % or median (interquartile range) unless otherwise indicated.

**Table 2:** Frequency and distribution of general health specific variables (*n* = 388).

Participant Characteristics	Frequency (*n*)	Percentage (%)
Smoking history		
Current	118	30.41%
Previous smoker	33	9.02%
Non-smoker	235	60.57%
**Alcohol use**		
Current	200	51.55%
Previous alcohol use	78	20.10%
No alcohol use	110	28.35%
**Alcohol misuse ^†^**		
CAGE score: 0–1	146	73.00%
CAGE score: 2–4	54	27.00%
**Illicit drug use**		
Current illicit/non-prescription drug use	11	2.84%
Previous illicit drug user	35	9.02%
No illicit drug use	342	88.14%
**Currently on treatment for other medical condition**	107	27.58%
**Substance use to manage work related stress (WRS)**		
Feel need to smoke to manage WRS	103	26.55%
Prescription drug use to manage WRS	65	16.75%
Feel need to drink alcohol to manage WRS	44	11.34%
Feel need to use illicit drugs to manage WRS	16	4.13%
**Ever diagnosed with a mental health condition**	43	11.08%
**Currently on treatment for mental health condition**	28	7.22%
**Family history of mental health condition**	44	11.34%

Data are presented as % or median (interquartile range) unless otherwise indicated. ^†^ Cage Score; Define positive score ≥2/4 positive responses to CAGE questions on problem drinking. WRS: work-related stress.

**Table 3:** Frequency and distribution of mental health specific variables (*n* = 388).

Participant Characteristics	Frequency (*n*)	Percentage (%)
**Prevalence of PTSD**		
All participants	118	30.41%
**Prevalence of PTSD by gender**		
Males (*n* = 213)	57	26.76%
Females (*n* = 175)	61	34.86%
**Prevalence of PTSD by job category/role**		
Operational staff (*n* = 277)	85	30.69%
Support staff (*n* = 111)	33	29.73%
**Role limitations due to emotional problems (SF36)**		
Any emotional problems with regular work in past 4 weeks	156	40.21%
Cut down on amount of time spent on work/other activities	94	24.23%
Accomplished less than would like	110	28.35%
Not working as carefully as usual	90	23.20 %
**Awareness of services to manage WRS**	277	71.39%
**Extent of training on how to manage WRS**		
No training	268	69.07%
Some training	110	28.35%
Extensive training	10	2.58%
**Extent of training on services available for WRS**		
No training	266	68.56%
Some training	111	28.61%
Extensive training	11	2.84%
**Barriers to seeking help for WRS**		
Fear that services are not confidential	149	38.40%
Fear that my career will be negatively affected	88	22.68%
Difficult to get time off from work	69	17.78%
Lack finances or medical aid	68	17.52%
Do not know where to get help	61	15.72%
Difficult to schedule appointment	48	12.37%
Lack transport to access help	42	10.82%
**How services/support for WRS can be improved**		
Train staff to recognize when stressed	262	67.52%
Have more supportive management	242	63.37%
Address staff shortages	243	62.63%
Provide counselling on premises	224	57.73%
Allow for debriefing or discussion	219	56.44%
Train supervisors to detect WRS	203	52.32%
Improve culture within service	154	39.70%
Provide accessible treatment services	153	39.43%
Provide group coaching	148	38.14%
Lessen workload	119	30.67%
Rotate shifts: work in high and low trauma settings	86	22.16%
Rotate shifts to allow enough rest	82	21.13%
Provide counselling telephonically	78	20.10%

Data are presented as % or median (interquartile range) unless otherwise indicated. PTSD: post-traumatic stress disorder; WRS: work-related stress.

**Table 4:** Descriptive results for preferred sources of support (*n* = 388).

Sources of Support	Likely to Seek Support
Spouse or partner (*n* = 336)	62.50%
Family member or friend (*n* = 345)	55.94%
Spiritual or religious leader (*n* = 337)	40.95%
Colleague or co-worker (*n* = 338)	29.29%
Telephonic counsellors (*n* = 340)	27.94%
Supervisor (*n* = 339)	25.96%
Employee assistance programme (*n* = 336)	25.60%
Occupational health/wellness staff (*n* = 336)	25.30%
Trade union or labour representative (*n* = 327)	20.18%

**Table 5:** Occupational and environmental risk factors for PTSD (Univariate and Bivariate analysis).

Risk Factors	Mean (SD)	Correlation with PTSD Score
SF36 QoL score (role limitation) *	37.2 (38.42)	−0.22 (*p* = 0.006)
Resilience (CD-RISC score)	28.0 (6.93)	−0.25 (*p* < 0.001)
Operational stress	33.8 (15.27)	0.56 (*p* < 0.001)
Organizational stress	42.5 (16.06)	0.46 (*p* < 0.001)
Chronic workplace stress ^†^	76.4 (28.41)	0.56 (*p* < 0.001)
Critical incident stress	27.7 (17.01)	0.34 (*p* < 0.001)
Posttraumatic stress	23.6 (22.41)	1

* SF36 Quality of Life score (role limitations due to emotional problems). ^†^ Chronic workplace stress (operational stress and organizational stress combined). CD-RISC: Connor–Davidson Resilience Scale.

**Table 6:** Unadjusted and adjusted regression analysis of the correlates of PTSD (N = 388).

	Unadjusted Univariate Analysis ^$^		Adjusted Multivariate Analysis ^$^ *	
Correlates	OR (95% CI)	*p*-Value	OR (95% CI)	*p*-Value
**Age** (*n* = 339)			-	-
≤30 years	1.00	1.00		
31–40 years	0.98 (0.53–1.82)	0.95		
41–50 years	1.19 (0.62–2.30)	0.60		
≥ 51 years	0.74 (0.30–1.84)	0.52		
**Gender**				
Male	1.00	1.00	-	-
Female	1.46 (0.95–2.26)	0.09	-	-
**Education**				
Basic (Grade 1 to 12)	1.00	1.00	-	-
Certificate	1.62 (0.71–3.69)	0.26	-	-
Diploma	0.84 (0.29–2.47)	0.76	-	-
Degree	2.03 (0.65–6.34)	0.23	-	-
**Smoking**				
Non-smoker	1.00	1.00	1.00	1.00
Ex-smoker	1.00 (0.46–2.20)	0.99	1.09 (0.46–2.59)	0.841
Current smoker	1.33 (0.83–2.14)	0.23	**1.76 (1.05–2.95)**	**0.033**
**Feel need to smoke to manage WRS**				
Never feel need	1.00	1.00	1.00	1.00
Feel need to smoke	**2.13 (1.33–3.41)**	**0.002**	**2.35 (1.40–3.93)**	**0.001**
**Alcohol misuse (*n* = 200)**				
No alcohol misuse (CAGE score 0–1)	1.00	1.00	1.00	1.00
Alcohol misuse (CAGE score 2–4)	**2.85 (1.50–5.44)**	**0.001**	**3.86 (1.80–8.23)**	**0.001**
**Feel need to use alcohol to manage WRS**				
Never feel need	1.00	1.00	1.00	1.00
Feel need to use alcohol	**3.55 (1.87–6.75)**	**< 0.001**	**6.37 (2.93–13.85)**	**<0.001**
**Drug/illicit substance use**				
Non-drug user	1.00	1.00	1.00	1.00
Ex-drug user	0.95 (0.44–2.06)	0.905	1.08 (0.48–2.41)	0.850
Current drug user	**4.18 (1.20–14.58)**	**0.025**	**16.4 (1.87–143.86)**	**0.012**
**Feel need to use illicit drugs to manage WRS**				
Never feel need	1.00	1.00	1.00	1.00
Feel need to use drugs	4.07 (1.44–11.48)	0.008	5.99 (1.74–20.62)	0.005
Feel need to use prescription drugs to manage WRS				
Never feel need	1.00	1.00	1.00	1.00
Feel need to use prescription drugs	**4.63 (2.65–8.09)**	**<0.001**	**4.51 (2.48–8.20)**	**<0.001**
**Job category (by department)**				
Managers, Admin, Finance & Other	1.00	1.00	1.00	1.00
Ambulance Services	3.10 (0.89–10.7)	0.075	2.97 (0.82–10.68)	0.096
HealthNet	**6 (1.32–27.10)**	**0.020**	5.02 (0.93–27.11)	0.061
Rescue Services	0.58 (0.09–3.8)	0.571	0.78 (0.11–5.40)	0.798
Call Centre	3.44 (0.95–12.54)	0.060	**4.04 (1.07–15.21)**	**0.039**
**Work location (within province)**				
Cape Town metropole	1.00	1.00	1.00	1.00
Rural areas	**0.61 (0.40–0.95)**	**0.027**	**0.90 (0.84–0.97)**	**0.006**
Mental health diagnosis				
Never diagnosed	1.00	1.00	1.00	1.00
Diagnosed	3.76 (1.96–7.21)	<0.001	3.52 (1.78–6.97)	<0.001
**Treatment for other medical condition**				
Not currently on treatment	1.00	1.00	1.00	1.00
Currently on treatment	**1.95 (1.22–3.11)**	**0.005**	**2.19 (1.29–3.73)**	**0.004**
**Emotional problems with regular work (past 4 weeks)**				
No emotional problems with regular work	1.00	1.00	1.00	1.00
Emotional problems with regular work	**5.37 (3.36–8.59)**	**<0.001**	**6.00 (3.57–10.10)**	**<0.001**
**SF36 QoL score (role limitation)**				
SF36 QoL score (≤3)	1.00	1.00	1.00	1.00
SF36 QoL score (4)	0.44 (0.18–1.07)	0.071	**0.33 (0.12–0.87)**	**0.024**
SF36 QoL score (5)	**0.33 (0.14–0.78)**	**0.012**	**0.26 (0.10–0.69)**	**0.007**
SF36 QoL score (6)	**0.38 (0.15–0.94)**	**0.036**	**0.34 (0.12–0.96)**	**0.042**
**Resilience (CD-RISC score)**				
CD-RISC score (≤26)	1.00	1.00	1.00	1.00
CD-RISC score (27–32)	**0.56 (0.34–0.91)**	**0.019**	0.67 (0.40–1.15)	0.148
CD-RISC score (33–40)	**0.26 (0.14–0.48)**	**<0.001**	**0.29 (0.15–0.57)**	**<0.001**
**Chronic workplace stress ^†^ (CSQ score)**				
CSQ score (≤63)	1.00	1.00	1.00	1.00
CSQ score (64–88)	**4.71 (2.16–10.30)**	**<0.001**	**4.46(1.93–10.31)**	**<0.001**
CSQ score (89–140)	**20.03 (9.33–43.03)**	**<0.001**	**19.83(8.75–44.89)**	**<0.001**
**Critical incident stress (CII score)**				
CII score (≤18)	1.00	1.00	1.00	1.00
CII score (19–37)	1.14 (0.64–2.03)	0.666	1.28 (0.66–2.48)	0.468
CII score (38–72)	**3.19 (1.88–5.40)**	**<0.001**	**4.27 (2.24–8.15)**	**<0.001**

* Data adjusted for age, gender and education.  ^$^ Statistically significant results indicated in bold ^†^ Chronic workplace stress (operational stress and organizational stress combined). CD-RISC: Connor–Davidson Resilience Scale. WRS: work-related stress.

## Data Availability

The data presented in this study are available on request from the corresponding author. The data are not publicly available due to legal and privacy issues.
